# Variation of human norovirus GII genotypes detected in Ibaraki, Japan, during 2012–2018

**DOI:** 10.1186/s13099-019-0303-z

**Published:** 2019-05-24

**Authors:** Takumi Motoya, Masahiro Umezawa, Aoi Saito, Keiko Goto, Ikuko Doi, Setsuko Fukaya, Noriko Nagata, Yoshiaki Ikeda, Kaori Okayama, Jumpei Aso, Yuki Matsushima, Taisei Ishioka, Akihide Ryo, Nobuya Sasaki, Kazuhiko Katayama, Hirokazu Kimura

**Affiliations:** 1 Ibaraki Prefectural Institute of Public Health, Ibaraki, Japan; 2 Faculty of Veterinary Medicine, Kitasato University, Aomori, Japan; 3 Gunma Paz University Graduate School of Health Science, Gunma, 370-0006 Japan; 4 Department of Respiratory Medicine, Kyorin University School of Medicine, Tokyo, Japan; 5 Kawasaki City Institute for Public Health, Kawasaki, Kanagawa Japan; 6 Takasaki City Public Health Center, Gunma, Japan; 7 Department of Molecular Biodefence Research, Yokohama City University Graduate School of Medicine, Kanagawa, Japan; 8 Laboratory of Viral Infection I, Kitasato Institute for Life Sciences, Kitasato University, Tokyo, Japan; 9 Infectious Disease Surveillance Center, National Institute of Infectious Diseases, Tokyo, Japan

**Keywords:** Epidemiology, Genotype, Norovirus, Outbreak, Viral load

## Abstract

**Background:**

Human norovirus (HuNoV) is the major cause of viral acute gastroenteritis for all age groups in various countries. HuNoV GII in particular accounted for the majority of norovirus outbreaks, among which GII.4 caused repeated outbreaks for a long time. Besides GII.4, other norovirus genotypes, GII.2, GII.6, and GII.17, have also been prevalent in various contexts in recent years, but few detailed epidemiological studies of them have been performed and are poorly understood. We thus conducted an epidemiological analysis of HuNoV GII in Ibaraki Prefecture, Japan, by performing surveillance in the six seasons from September 2012 to August 2018.

**Results:**

HuNoV GI occurred almost sporadically for all genotypes; however, each genotype of GII exhibited its typical epidemiological characteristics. Although the number of outbreaks of GII.4 decreased season by season, it reemerged in 2017/2018 season. The timing of the epidemic peak in terms of number of cases for GII.17 differed from that for the other genotypes. The patients age with GII.2 and GII.6 were younger and outbreak of GII.17 occurred frequently as food poisoning. Namely, the primarily infected outbreak group differed for each genotype of HuNoV GII. Moreover, the viral load of patients differed according to the genotype.

**Conclusions:**

Various HuNoV genotypes including GII.2, GII.4, GII.6, and GII.17 were shown to be associated with various types of outbreak sites (at childcare and educational facilities, involving cases of food poisoning, and at elderly nursing homes) in this study. These genotypes emerged in recent years, and their prevalence patterns differed from each other. Moreover, differences in outbreak sites and viral load of patients among the genotypes were identified.

## Background

Human norovirus (HuNoV) belongs to the family Caliciviridae, genus *Norovirus*. It is a leading causative agent of acute gastroenteritis in people of all ages [1]. Many reports have suggested that the HuNoV genome can evolve rapidly, resulting in many different genotypes [2, 3]. At present, HuNoV is further classified into two genogroups (genogroups I and II) and over 30 genotypes (GI.1–GI.9 and GII.1–GII.22), as revealed by detailed genetic analyses of the capsid gene [4].

Previous molecular epidemiological studies showed that some genotypes of HuNoV, including GII.2, GII.4, GII.6, and GII.17, were particularly prevalent in gastroenteritis cases worldwide during the last 10 years [5–7]. Among these, GII.4 variant strains suddenly emerged and caused pandemics of gastroenteritis in many regions including Japan during 2006–2014 [4, 8]. This genotype has been associated with not only gastroenteritis in infants but also food poisoning in adults in various countries [9], while the prevalence of the virus may have declined during the last three seasons [10–12]. Another new genotype, GII.P17–GII.17, also suddenly emerged and caused large outbreaks in some countries [13], including large food poisoning-related outbreaks in Japan [14]. Furthermore, GII.2 variant strains reemerged in the 2016/2017 season and caused pandemics in various countries including Germany, China, and Japan [15–17]. However, there is still no comprehensive understanding of the local molecular epidemiology of HuNoV in Japan.

Therefore, we studied the relationships between the prevalent NoV genotypes associated with gastroenteritis outbreaks and epidemiologic data in Ibaraki Prefecture, Japan, during the 2012–2018 seasons to better understanding the molecular epidemiology in a domestic area.

## Results

### Relationships among HuNoV genotype, season, patient age, and outbreak site of infection

**Fig. 1:**
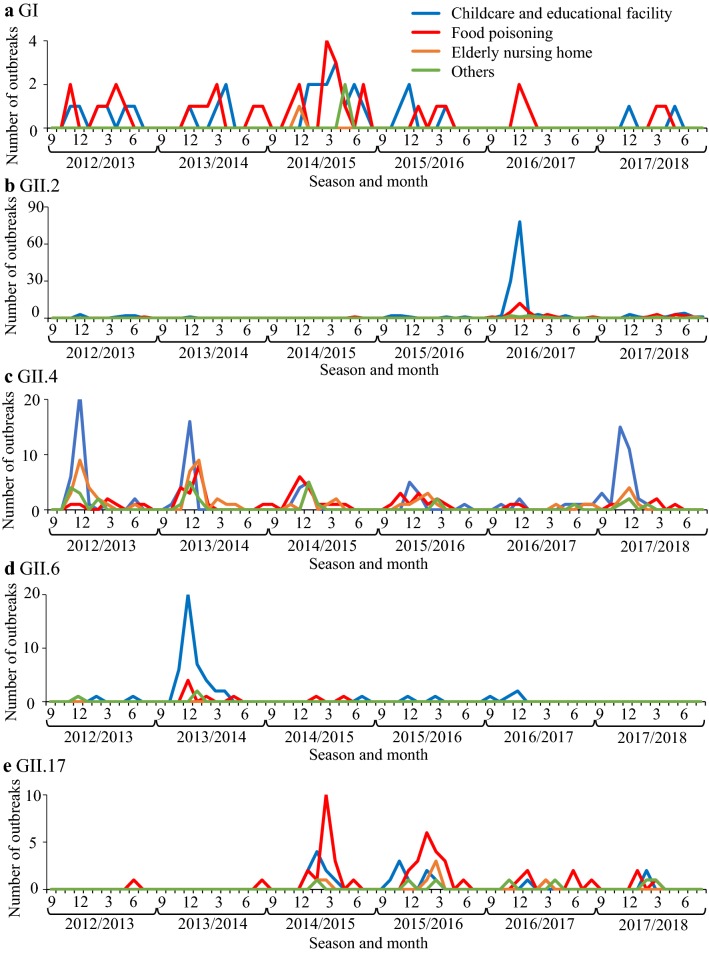
Relationship between the occurrence of outbreaks for each genotype of NoV and the site of infection. The number of outbreaks of **a** GI, **b** GII.2, **c** GII.4, **d** GII.6, and **e** GII.17 is shown as a line graph. Blue indicates cases at childcare and educational facilities, red indicates cases involving food poisoning, orange indicates cases at elderly nursing homes, and green indicates others. The vertical axis shows the number of outbreaks, and the horizontal axis shows the season and month of appearance

A total of 4588 clinical fecal specimens collected from September 2012 to August 2018 were examined to detect HuNoV (Table 1). Among these, HuNoV GI was detected in 244 specimens (around 5% of all specimens) and HuNoV GII in 2437 (around 53%). Detailed data on the seasonal variations, detected genotypes, and the outbreak site are shown in Tables 2 and 3 and Fig. 1. First, during the 2012–2015 seasons, GII.4 was detected in many cases in all patient sites, including outbreaks at childcare (0–6 years old), educational facilities (6–15 years old), and elderly nursing homes, and cases involving food poisoning. The GII.2 was the main genotype detected in outbreaks at childcare and educational facilities in the 2016/2017 season. GII.6 was mainly detected in the 2013/2014 season in outbreaks at childcare and educational facilities. GII.17 was suddenly detected in outbreaks involving food poisoning from the 2014/2015 season. GII.4 reemerged and caused outbreaks at childcare and educational facilities during the 2017/2018 season. In addition, GII.2, GII.4, and GII.6 were detected during September–March, whereas GII.17 was detected during January–April (Fig. 1). Finally, the GI virus was mainly detected in the 2014/2015 season from outbreaks at childcare and educational facilities and from cases involving food poisoning (Table 2). Moreover, unlike GII virus, GI virus was sporadically detected throughout the seasons (Fig. 1). These results suggest that various types of HuNoV were associated with the outbreaks of gastroenteritis in Ibaraki Prefecture.

**Table 1:** Detected viruses in this study

Season	2012/2013	2013/2014	2014/2015	2015/2016	2016/2017	2017/2018	Total	Rate (%)
Samples	560	876	842	694	908	708	4,588	
NoV GI	36	40	129	18	3	18	244	5.3
NoV GII	268	471	347	332	631	388	2,437	53.1
GII.2	35	7	1	35	493	112	683	14.9
GII.4	186	262	187	108	53	234	1,030	22.4
GII.6	15	161	4	7	13		200	4.4
GII.17	1	19	96	108	34	21	279	6.1
Other GII	31	22	59	74	38	21	242	5.3
RVA	28	36	5	50	7	7	133	2.9
SaV	20	13	37	51	16	62	199	4.3
AdV	1	12	7	8	9	16	53	1.2
AstV			5	1	3		9	0.2

*RVA* rotavirus group A, *SaV* sapovirus, *AdV* adenovirus, *AstV* astrovirus

**Table 2:** Detected genotypes of GI in each situation

Season	2012/2013	2013/2014	2014/2015	2015/2016	2016/2017	2017/2018	Total
GI	13	11	29	7	3	4	67
C	6	4	13	4		2	29
F	7	7	13	3	3	2	35
E			1				1
O			2				2
GI.2	1	3	10	4	0	0	18
C	1	2	4	2			9
F		1	5	2			8
E							
O			1				1
GI.3	4	2	17	2	0	2	27
C			8	2		1	11
F	4	2	7			1	14
E			1				1
O			1				1
GI.4	0	3	1	0	0	1	5
C						1	1
F		3	1				4
E							
O							
GI.6	7	2	0	1	0	0	10
C	5	1					6
F	2	1		1			4
E							
O							
Other GI	1	1	1	0	3	1	7
C		1	1				2
F	1				3	1	5
E							
O							

*C* childcare and educational facility, *F* food poisoning, *E* elderly nursing home, *O* others

**Table 3:** Detected genotypes of GII in each situation

Season	2012/2013	2013/2014	2014/2015	2015/2016	2016/2017	2017/2018	Total
GII	85	122	92	83	189	89	660
C	46	67	25	36	135	52	361
F	9	25	48	31	40	22	175
E	20	20	11	12	4	8	75
O	10	10	8	4	10	7	49
GII.2	9	1	1	7	152	27	197
C	8	1		7	117	15	148
F	1		1		28	10	40
E						1	1
O					7	1	8
GII.4	67	66	43	31	15	52	274
C	31	21	10	9	7	33	111
F	7	17	18	12	4	8	66
E	20	20	9	8	3	7	67
O	9	8	6	2	1	4	30
GII.6	4	49	3	2	4	0	62
C	3	41	1	2	4		51
F		6	2				8
E							
O	1	2					3
GII.17	1	1	29	33	11	7	82
C			9	8	1	2	20
F	1	1	17	19	7	3	48
E			2	4	1		7
O			1	2	2	2	7
Other GII	4	5	16	10	7	3	45
C	4	4	5	10	6	2	31
F		1	10		1	1	13
E							
O			1				1

*C* childcare and educational facility, *F* food poisoning, *E* elderly nursing home, *O* others

### Relationships among age, viral load, and HuNoV genotype in the fecal specimens

In the present study, we analyzed the relationships among age, viral load, and HuNoV genotype in the fecal specimens. The ages of the patients were significantly lower in whom GII.2 and GII.6 were detected than in whom GI, GII.4, and GII.17 were detected. Moreover, the age of patients in whom GII.6 was detected was significantly lower than for GII.2 (Table 4). We also analyzed the viral loads for some genotypes, including GI, GII.2, GII.4, GII.6, and GII.17, using real-time (RT)-PCR in the fecal specimens (Table 5). The HuNoV genome copy numbers of GII.2 were significantly higher than that of GI, GII.4, GII.6, and GII.17. These results suggested that the patients with GII.2 excreted more viruses than those infected with viruses of other genotypes.

**Table 4:**
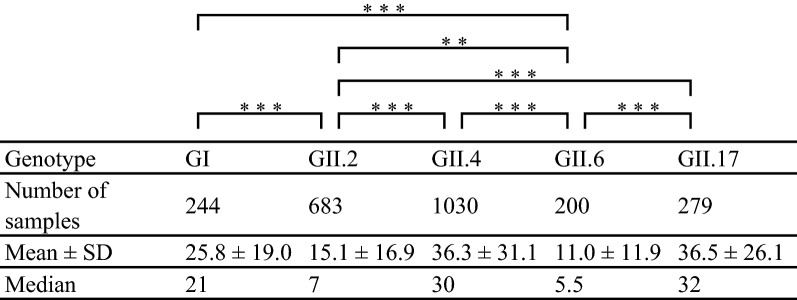
The patient age of each norovirus genotype

The asterisks represent *p*-values as follows: * *p* < 0.05, ** *p* < 0.01, *** *p* < 0.001

**Table 5:**
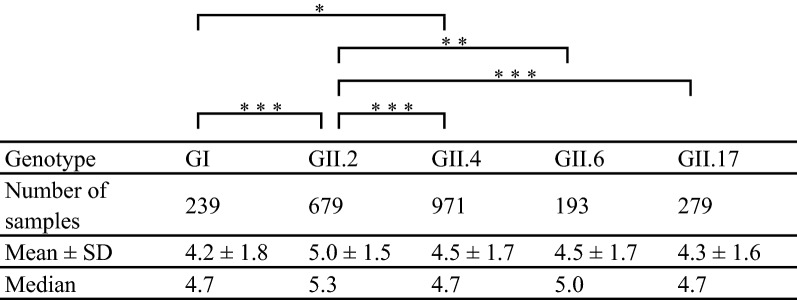
Viral load (log_10_) in the patient of each norovirus genotype

The asterisks represent *p*-values as follows: * *p* < 0.05, ** *p* < 0.01, *** *p* < 0.001

### Phylogeny of the detected HuNoV viruses

**Fig. 2:**
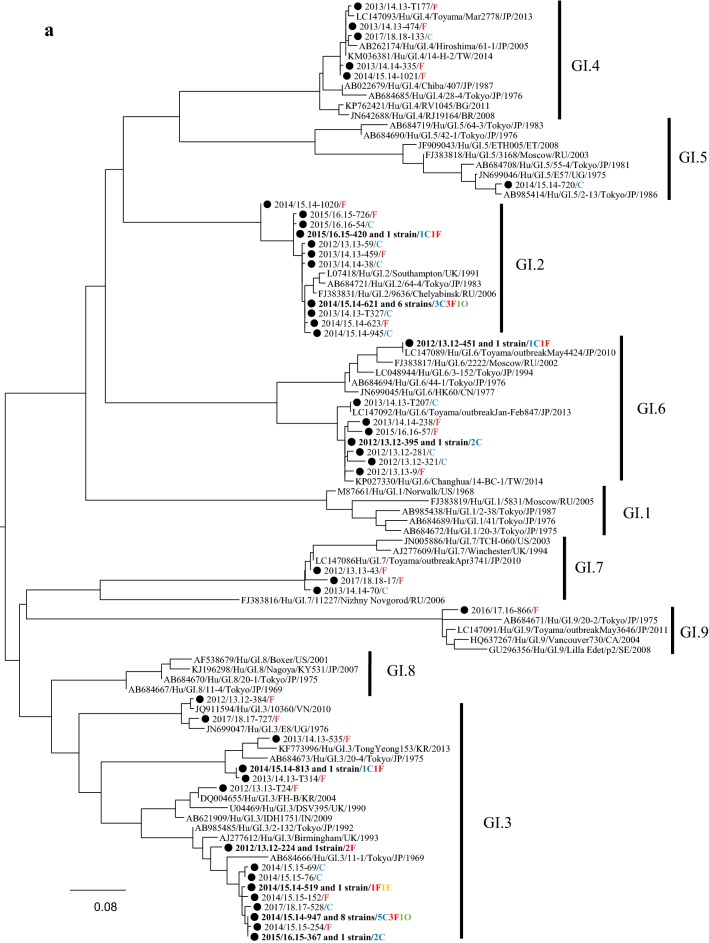

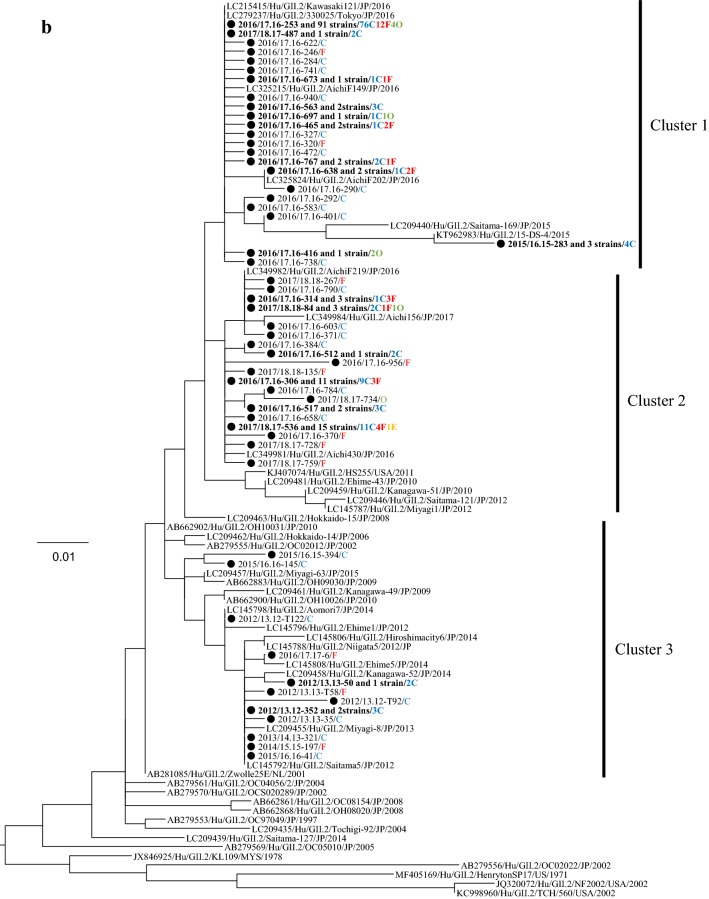

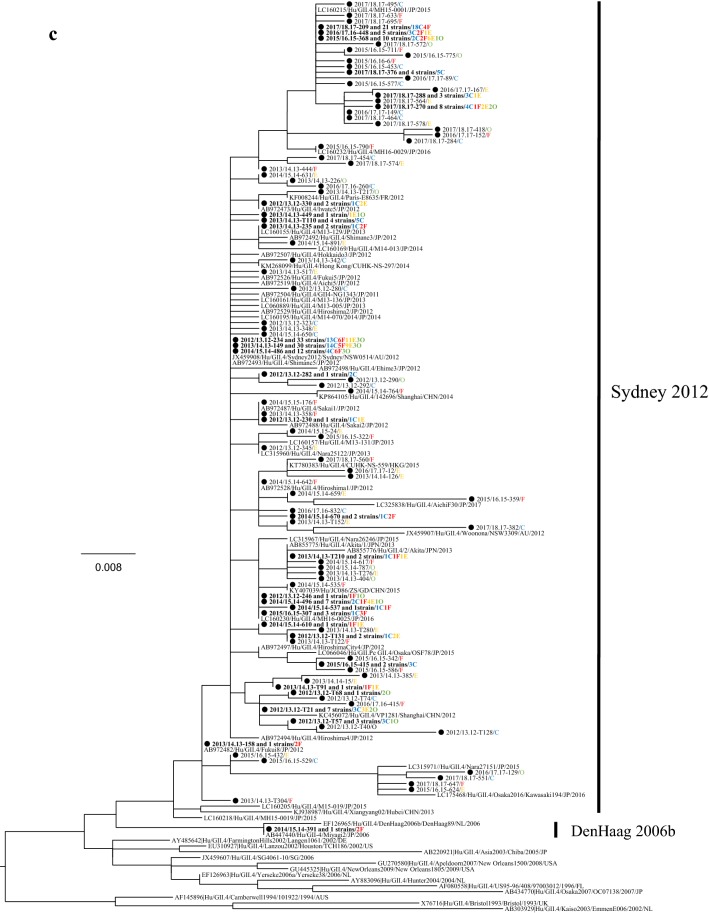

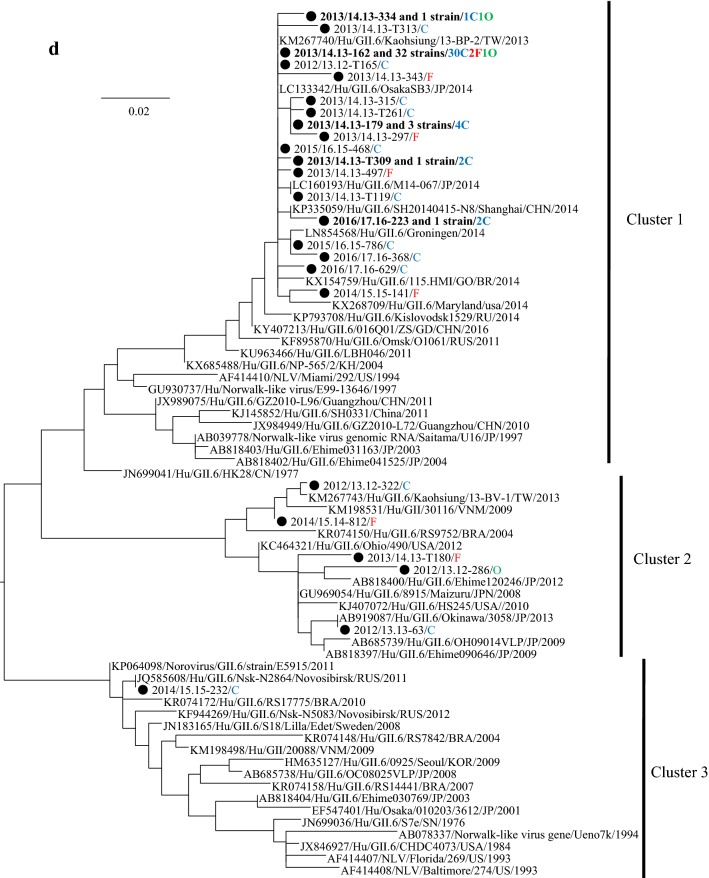

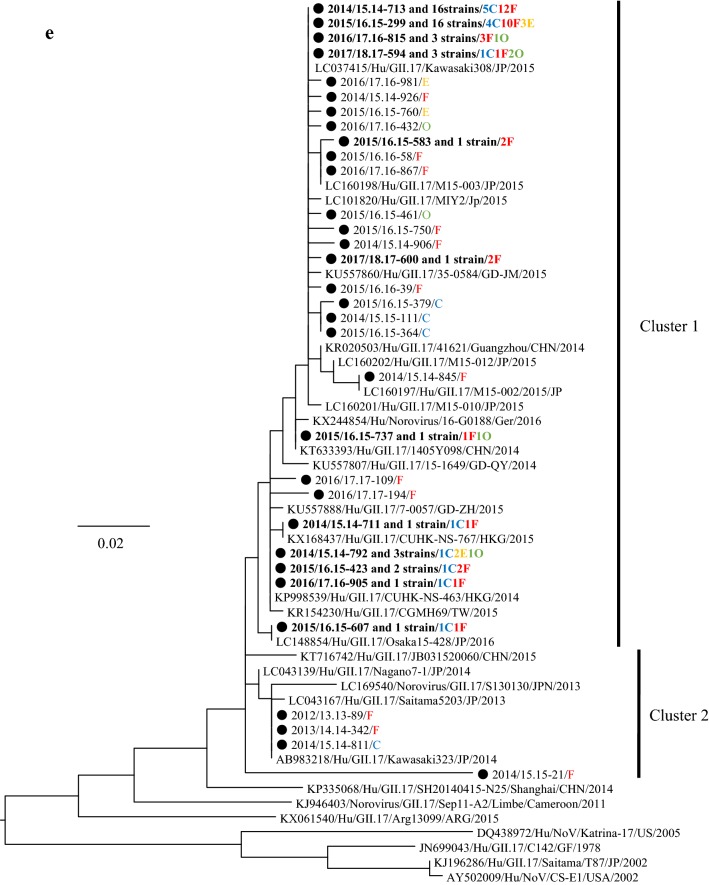
Gene phylogenetic tree was created by the ML method using 295 or 298 nt (GI) and 282 nt (GII) from the 5′-end of *VP1*. **a** Gene phylogenetic tree of GI. Nucleotide substitution model was GTR + Gamma. **b** Gene phylogenetic tree of GII.2. Nucleotide substitution model was K80 + Gamma. **c** Gene phylogenetic tree of GII.4. Nucleotide substitution model was K80 + Gamma. **d** Gene phylogenetic tree of GII.6. Nucleotide substitution model was K80 + Gamma. **e** Gene phylogenetic tree of GII.17. Nucleotide substitution model was GTR + Invariant. The strains detected in this study were shown as a black circle. Strains detected in multiple cases are shown in bold. The site of the outbreaks and their number are described at the end of the strain name. Cases at childcare and educational facilities (C) are colored blue, cases involving food poisoning (F) are colored red, those at elderly nursing homes (E) are colored orange, and others (O) are colored green

We performed a phylogenetic analysis based on the *VP1* gene sequences of GI and genotypes of GII; GII.2, GII.4, GII.6, and GII.17 using the maximum likelihood (ML) method (Fig. 2a–e). First, 7 genotypes of GI virus such as GI.2, 3, 4, 5, 6, 7, and 9 were detected in this study (Fig. 2a). The tree of the genotype GII.2 formed three major clusters (Fig. 2b). GII.2 strains belonging to Clusters 1 and 2 were the main ones detected during the last two seasons. In the present tree, GII.2 virus detected in the 2016/17 season were classified into the Clusters 1 and 2, whereas the GII.2 virus mainly detected in the 2017/18 season were classified into Cluster 2. The GII.4 strains formed many clusters, although almost all of them were classified into the Sydney 2012 type (Fig. 2c). Among them, the GII.4 virus detected during the 2012–2015 seasons were genetically identical with a GII.4 prototype strain (accession no. JX459908), whereas the virus detected in some cases during 2015–2018 seasons were genetically identical with another prototype GII.4 (accession no. LC160215). GII.6 strains formed three clear clusters. Among these, the strains detected in the 2013/2014 season belonged to Cluster 1, whereas the strains detected in the 2012/2013 season mainly belonged to Cluster 2 (Fig. 2d). Most GII.17 strains belonged to Cluster 1 (Kawasaki308 type), whereas some strains belonged to Cluster 2 (Kawasaki323 type) (Fig. 2e). In the present cases, we detected genotype GII.17 in some cases during the 2014–2018 seasons, and these strains were genetically identical with a prototype of GII.17 virus (Kawasaki 308 strain). These results suggest that various genotypes of GII viruses have been associated with a range of outbreaks in Ibaraki Prefecture.

### Pairwise distance of the strains

**Fig. 3:**
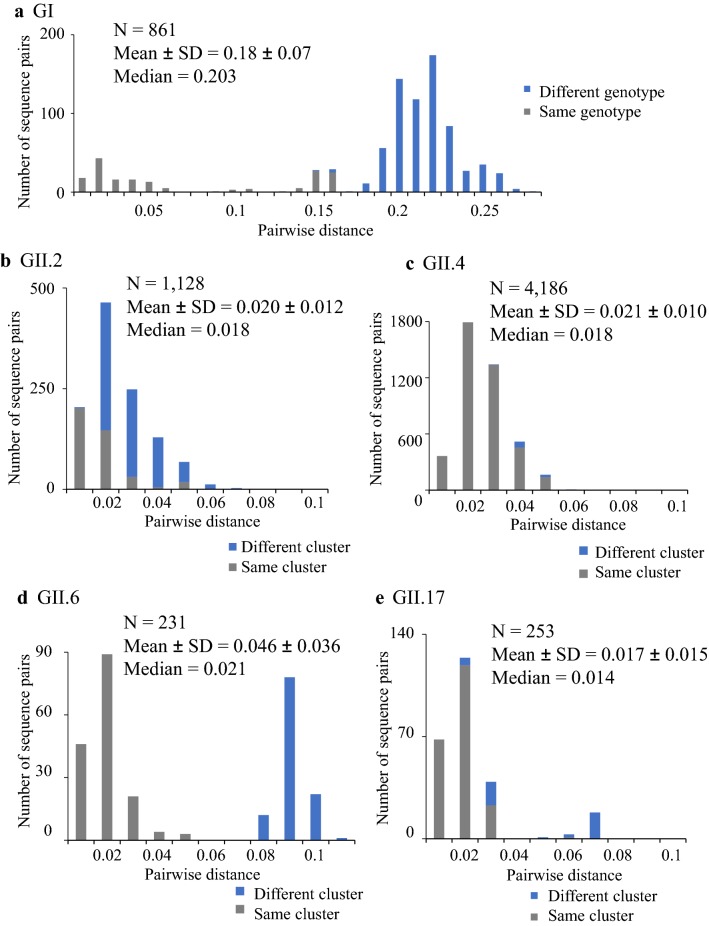
Distributions of the pairwise distance values of partial *VP1* gene of NoV detected in Ibaraki Prefecture. **a** GI was analyzed for a total of 42 strains. **b** GII.2 was analyzed for a total of 48 strains. **c** GII.4 was analyzed for a total of 92 strains. **d** GII.6 was analyzed for a total of 22 strains. **e** GII.17 was analyzed for a total of 23 strains. The vertical axis shows the number of sequence pairs, and the horizontal axis shows the pairwise distance. Blue indicates different clusters, and gray indicates the same cluster

To analyze the genetic divergence of the present strains, we calculated the pairwise distances of the GI, GII.2, GII.4, GII.6, and GII.17 strains (Fig. 3a–e). First, the pairwise distance value among the GI strains was 0.18 ± 0.07 (mean ± standard deviation [SD]), and the intra-genotypic pairwise distance value was 0.067 ± 0.061 (mean ± SD). The intra-genotypic pairwise distance value of the present GII.2 was 0.020 ± 0.012 (mean ± SD), whereas that of the GII.4, GII6, and GII.17 values were 0.021 ± 0.010 (mean ± SD), 0.046 ± 0.036 (mean ± SD), and 0.017 ± 0.015 (mean ± SD), respectively. Overall, these genetic distances were relatively short within the same cluster. Thus, the results suggest that the strains analyzed here had not undergone wide genetic divergence.

## Discussion

In this study, we performed a molecular epidemiological study of HuNoV infection in Ibaraki Prefecture, Japan, during the 2012–2018 seasons. The main findings were as follows: (i) various HuNoV genotypes including GII.2, GII.4, GII.6, and GII.17 were associated with the outbreaks of gastroenteritis in Ibaraki Prefecture; (ii) the GII.2-infected subjects showed a higher viral load in fecal specimens than those infected with viruses of other genotypes; and (iii) the detected strains had relatively low genetic divergence.

It has been reported that, although other GII genotypes were previously prevalent, GII.4 Den Haag 2006b suddenly emerged and caused pandemics in the 2006/2007 season [18, 19]. Moreover, variants (Den Haag 2006b type, New Orleans 2009 type, and Sydney 2012 type) of the GII.4 caused many outbreaks up to the 2013/2014 season [8]. However, after the 2014/2015 season, not only was GII.4 prevalent but also other genotypes, such as GII.2 and GII.17, were associated with outbreaks [13, 20]. A possible reason for the alterations of the prevalent GII genotypes; is that acquired herd immunities due to large outbreaks may affect human population [6, 21, 22]. Overall, the identified trends regarding the prevalent genotypes in Ibaraki Prefecture are compatible with those in other reports [13, 17, 23–27].

Previous studies have demonstrated that the GII virus was frequently detected compared with the GI virus from the NoV infection [28–31], which was consistent with our results. In contrast, both the GI and GII virus genomes were detected in environmental water at equivalent frequencies using real-time RT-PCR [32, 33] possibly due to the difference in stability between the GI and GII capsid proteins [34]. Indeed, Pogan et al. [34] showed that, unlike the GII.17 virus, the GI.1 virus may not be stable at high pH (over pH 8) using virus-like particles; however, this study did not examine the infectivity. We speculate that the stabilities of the virus particles between GI and GII viruses reflect the infectivity of these viruses to humans.

The phylogenetic tree of GII.4 created here showed that almost all detected strains were of the Sydney 2012 type, although these strains formed many small clusters in the tree (Fig. 3c). Previous reports have suggested that GII.4 suddenly emerged and caused pandemics of gastroenteritis in the 2006/2007 season (Den Haag 2006b type) and that some GII.4 variants such as Osaka 2007, Apeldoorn 2007, New Orleans 2009, and Sydney 2012 were subsequently generated [4, 35–37]. Among these, Sydney 2012 type caused as many pandemics of gastroenteritis as Den Haag 2006b type [4, 35]. The results suggested that the GII.4 Sydney variant was also associated with gastroenteritis outbreaks at childcare and educational facilities, in cases of food poisoning, and at elderly nursing homes. This finding is compatible with the previous reports [36, 38], which suggested that GII.4 was the most dominant type during the 2006–2014 seasons, whereas a small number of GII.2 were detected in this period [20, 39, 40]. However, GII.2 was the most prevalent type in the 2016/2017 season in various countries, including Germany, France, USA, China, and Japan [16, 17, 25, 26]. In Ibaraki Prefecture, GII.2 was also detected from many outbreaks in the 2016/2017 season associated with childcare and educational facility. In the phylogenetic tree created here, distinct clusters were formed by the GII.2 strains detected in the 2016/2017 season and those from other seasons [20, 40]. It is suggested that the GII.2 strains detected in the 2016/2017 season were recombinant, which is compatible with the findings from very recent studies [16]. Although we did not examine the polymerase type of the present GII.2 strains, such recombination may have been associated with the prevalence of GII.2 in Ibaraki Prefecture. Next, GII.17 was detected from the 2013/2014 season onwards, which was associated with many food poisoning outbreaks in Ibaraki (Table 3). Moreover, the periods of greatest prevalence differed between GII.17 and other genotypes such as GII.2, GII.4, and GII.6 (Fig. 1). The reason for this is not understood, but this finding is also compatible with previous reports [41, 42].

Next, we examined the viral loads among infections with viruses of various genotypes including GII.2, GII.4, GII.6, and GII.17. The results showed that the viral loads of GII.2 were higher than for the other genotypes and the age of patients infected with HuNoV GII.6 was lower than for the other genotypes. Previous reports suggested that the HuNoV viral loads in feces are associated with the age and immunity status of the hosts, although the reasons for this are not known [43, 44]. Although there are few previous reports describing the viral load of HuNoV, the propagation rate of GII.2 may not be higher than that of other genotypes [45, 46]. A possible reason for this is that we did not examine the differences in propagation among the genotypes and did not take into account the number of days since the patient had developed symptoms in this study. Moreover, the methods used in this study possibly cannot be used to analyze samples containing low numbers of the NoV genomes (approximately > 10 copies/experiment). However, if samples contain large numbers of genomes of different NoV genogroups, we may be able to analyze genotypes of plural NoV genogroup in each sample. In this study, we could analyze plural NoV genogroups in 23 samples of 19 cases. Currently, Next Generation Sequencing is expensive; therefore, in this study, we used conventional methods to analyze the samples as previously described [47]. Thus, further studies may be needed to clarify the epidemiology of HuNoV.

## Conclusions

We showed in this study that many HuNoV genotypes, including GII.2, GII.4, GII.6, and GII.17, were associated with various types of outbreak sites (at childcare and educational facilities, in cases of food poisoning, and at elderly nursing homes) in this study. These genotypes emerged in recent years, and they exhibited distinct patterns of prevalence. Moreover, differences in the outbreak sites and viral load of patients were identified among the genotypes. To better understand the molecular epidemiology of HuNoV infection, ongoing molecular epidemiological studies may be needed.

## Methods

### Sample collection

Fecal specimens and patient information were collected for the following two types of outbreak cases among the surveillance system in Ibaraki Prefecture in Japan: (1) group cases that suspected outbreaks of human-to-human infectious disease and (2) group cases that suspected outbreaks of foodborne infectious disease. Public health centers collected information and specimens from the patients in both cases. In this study, we targeted population outbreak patients; therefore, whether the patients were administered to hospitals remains unknown. Patients without data on sex and age were omitted, and a total of 4588 specimens were collected by surveillance in the six seasons from September 2012 to August 2018 (Table 1).

### Epidemiological data analyses

For each genotype, we compared and considered the epidemiological data of specimens positive for HuNoV GII (season, age group, viral load, and site of infection). Infection cases were classified into the following four groups regarding the site of infection as well as the age of the patients: (1) kindergarten, nursery school, and primary school (childcare and educational facilities: C), (2) suspected food poisoning (F), (3) elderly nursing homes (E), and (4) others (O).

In this study, food poisoning was defined as the outbreaks of the gastroenteritis due to foods served for commercial purposes from the food provision facility.

### Detecting norovirus GII, sequencing, and genotyping

Fecal specimens were adjusted to 10 wt% with phosphate-buffered saline and centrifuged at 10,000×*g* for 10 min at 4 °C. The nucleic acids were extracted from the supernatant using QIAamp Viral RNA Mini Kit (Qiagen). Subsequently, complementary DNA (cDNA) was prepared by reverse transcription using PrimeScript™ RT Reagent Kit (Perfect Real Time) (Takara Bio). It was then used for quantitative polymerase chain reaction (q-PCR), which was performed using the TaqMan probe PCR system as described previously [48].

All RNA for which HuNoV GI and GII were determined to be positive by q-PCR was amplified using the PrimeScript™ II High Fidelity One Step RT-PCR Kit (Takara Bio) with G1SKF/G1SKR and G2SKF/G2SKR primers, respectively [47]. The nucleic acid sequence of the PCR product was determined by direct sequencing using the BigDye Terminator v3.1 Cycle Sequencing Kit (Thermo Fisher Scientific). The resulting sequence was genotyped using the Norovirus Genotyping Tool [49]. If the genotypes were the same among samples collected in the same case, one sequence was selected, and a dataset of the gene sequence was prepared.

### Calculation of pairwise distance

We analyzed pairwise distances to assess the genetic distances between human GII strains detected in Ibaraki Prefecture. Among the viral genes, 100% matched strains were omitted and pairwise distance values were calculated using MEGA 6 [50].

### Phylogenetic tree analysis

The obtained gene sequence was compiled for each genotype, and a dataset was obtained by adding standard strains. We revealed the nucleotide substitution model with KAKUSAN 4 [51] and performed a phylogenetic tree analysis using the maximum likelihood method with MEGA 6 [50]. The strains detected in this study are indicated as a black circle. When 100% homologous sequence strains were detected in the same season, only one strain was retained and indicated in bold; the other sequence(s) was omitted from the dataset. The sites regarding the outbreaks and their number are described at the end of the strain name. In addition, cases involving childcare and educational facilities (C) are colored blue, those involving cases of food poisoning (F) are colored red, those at elderly nursing homes (E) are colored orange, and others (O) are colored green.

### Statistical analysis

Statistical analysis was performed using EZR software [52]. After conducting the Kruskal–Wallis test as a statistical analysis on the age and viral load distribution of patients in each genotype, Holm’s multiple comparison test was performed.

## Data Availability

All data generated or analyzed during this study are included in this published article (and its Additional files).

## Abbreviations

HuNoV: human norovirus
GI: genogroup I
GII: genogroup II
PCR: polymerase chain reaction
cDNA: complementary DNA
q-PCR: real-time polymerase chain reaction
ML: maximum likelihood
